# Activated carbon, a useful medium to bind chlordecone in soil and limit its transfer to growing goat kids

**DOI:** 10.1371/journal.pone.0179548

**Published:** 2017-07-19

**Authors:** Sarah Yehya, Matthieu Delannoy, Agnès Fournier, Moomen Baroudi, Guido Rychen, Cyril Feidt

**Affiliations:** 1 Université de Lorraine, INRA USC 340, UR AFPA, 2 avenue de la Forêt de Haye TSA, Vandœuvre-lès-Nancy, France; 2 Lebanese University–Faculty of Public Health-Section III, L.S.E.E., Tripoli, Lebanon; RMIT University, AUSTRALIA

## Abstract

Chlordecone (Kepone) (CLD) is a highly persistent pesticide which was extensively used in the French West Indies; high levels of CLD can still currently be found in large agricultural areas. As CLD transfers from soil to animals mainly via involuntary ingestion, the consumption of foodstuffs derived from animals raised in contaminated areas may significantly contribute to exposure of humans to CLD. The present study was designed to test the efficacy of two different activated carbons (ACs) sources in limiting CLD transfer from soil to animal. Three soils (ASs) were prepared according to the OECD guideline 207. One standard soil (SS) lacking AC, and two modified preparations of SS supplemented with 2% coconut-based activated carbon (ORBO), SSO or with 2% lignite-based one (DARCO), SSD. All three soils were spiked with 10 μg of kepone per g of dry matter and aged for three weeks. This study involved 15 goat kids randomly assigned to the 3 experimental groups (n = 5/group), which were fed the experimental matrices at an exposure dose of 10 μg CLD per kg of body weight per day. After 21 d of oral exposure, CLD in adipose tissue and liver were analysed by LC-MS-MS. A significant decrease of 63.7% and 74.7% of CLD concentrations in adipose tissue and liver, respectively, were obtained from animals exposed using SS containing DARCO as compared to those receiving only SS. Decreases in CLD levels of 98.2% (adipose tissue) and 98.7% (liver) were obtained for animals exposed using SS containing ORBO. This study leads us to conclude that (i) the presence of AC in CLD-contaminated soil strongly reduces CLD bioavailability, and (ii) the efficacy depends on the nature and characteristics of the AC used.

## 1. Introduction

Chlordecone (CLD) is a chlorinated polycyclic ketone pesticide recognized as a Persistent Organic Pollutant (POP). The use of this pesticide against the banana black weevil (*Cosmopolites sordidus*) in Martinique and Guadeloupe (French West Indies) resulted in the spread of large amounts of this pesticide before its formal ban in 1993 in France. Since this pollutant is highly retained and persistent in soil, superficial layers of large areas of agricultural soils are extremely contaminated (>1mg/kg) [1]. This contamination is believed to last even up to several centuries [2]. In addition, its lipophilic properties (log Kow = 4.5 [3]) and high affinity for animal tissues leads to significant accumulation of this pesticide in animals [4–7]. As a consequence, a major human health concern related to CLD transfer from soil to animals has been raised, particularly in regards to local animal food production. Strategies to avoid contamination of outdoor reared animals are therefore urgently needed.

CLD-transfer to lambs [8], piglets [9] and laying hens [10] have been proven to not be limited by the type and properties of Antillean soils (nitisol or andosol). The specific efficiency of this transfer is related to the availability of this pesticide for outdoor reared animals. A means to reduce the soil bound CLD availability and its further potential absorption could be by the sequestration of this pollutant into a non-absorbable shape. Activated carbon (AC) is known as a potential medium for sequestration of many organic pollutants [11–14], and is already used to decontaminate water in the French West Indies [15]. Thus, *in-situ* sequestration using AC could be a promising means to limit the CLD-transfer from soil to animal.

The present study was conducted to assess the potential of two different ACs to efficiently limit CLD transfer from soil to young ruminants.

## 2. Material and method

### 2.1. Experimental design

The experimental design was aimed towards comparing CLD bioavailability in CLD-contaminated soils supplemented or not with two different AC sources derived from lignite (DARCO) or from coconut shell (ORBO 32).

**Fig 1:**
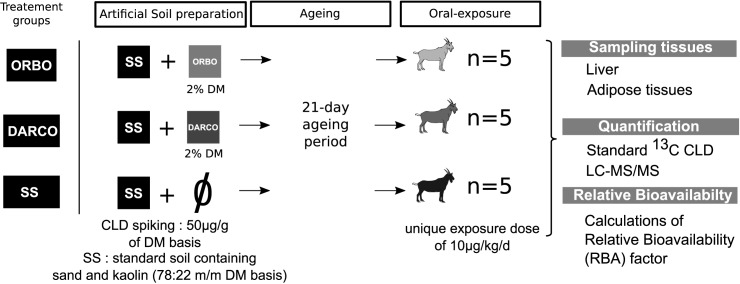
Experimental design of the present study.

Three groups of randomly assigned goat kids (n = 5 per group) were fed either one of these 3 soils. During the exposure period (day1 to day 21), kids received daily a moistened dough ball with one of the experimental soils for an exposure dose of 10 μg of CLD per kg of body weight. At the end of the contamination period (day 21), the animals were euthanized in order to collect the perirenal adipose tissue and the liver. Concentrations of CLD in those tissues were determined using LC-MS/MS. The Relative BioAvailability (RBA) factors of soil bound CLD were estimated using concentrations of CLD in the adipose tissue and in liver [16,17]. The experimental design of the present study is depicted in Fig 1.

### 2.2. Animals, ethics and housing

Fifteen 4-month-old Alpine male kids (*Capra hircus*, from Laneuvelotte, France) were used. Animals were allowed a 21-day acclimation period before starting the exposure period. Each experimental group (n = 5) was kept in a cage covered with straw and equipped with individual feeders in the URAFPA animal facilities (Université de Lorraine, Vandœuvre-les-Nancy, France). Temperature was maintained between 20–21°C. All animals were individually weighed every 2 days. Daily ingestion was estimated by subtracting leftovers from provided rations. Water was provided *ad libitum* by nipple waterers throughout the entire study. This project was approved by the institutional committee for ethical use of animal experimentation (Authorization number: 00270.02, authorized by the French Ministry of Higher Education and Research) and was carried out in strict accordance with the principles established in the Guide for the Care and Use of Laboratory Animals of the French Ministry of Agriculture, Agrifood, and Forestry and European Council Directive (European directive 2010/63/EU).

### 2.3. Dosing material and exposure methodology

#### 2.3.1. Activated carbon characteristics

Surfaces Specific Areas (SSA) were measured at the LIEC laboratory (Vandoeuvre-lès-Nancy, France) using the 77K Nitrogen adsorption method and performed on a Belsorp-mini II device (BELJAPAN, Inc Chemicals, JAPAN). The BET method was then used to estimate the SSA, using a 16.3 Å cross-sectional area of nitrogen molecules [18]. The De Boer method was carried out to determine external surface area.

DARCO displays an SSA of 793.8 ± 14.5 m2.g^-1^ and ORBO an SSA of 1126.3 ± 11.9 m^2^.g^-1^. Microporous surfaces represented 56% and 80% of the overall porosity for DARCO and ORBO respectively. Chemicals used and preparation of soils are described in Table 1.

**Table 1:** Composition of the different artificial soils and treatment of the experiment.

	Sand	Kaolin	Activated carbon	Activated carbon	Kepone	Time of maturation
	See Sand (Carl Roth GmbH, Karlsruhe, Germany)	(Sigma-aldrich, St Louis, USA)	DARCO ® (Sigma-aldrich, St Louis, USA)	ORBO 32 ® (Sigma-aldrich, St Louis, USA)	Concentration (μg.g^-1^ of DM) (Sigma-aldrich, Supelco)	in days
Standard soil (SS)	77.8%	22.2%	-	-	100	21
SS with DARCO (AC)	76.2%	21.8%	2%	-	100	21
SS with ORBO (AC)	76.2%	21.8%	-	2%	100	21

Percentages are DM basis of artificial soil.

#### 2.3.2. Soil preparation and spiking technique

Three artificial soils were prepared as described in Table 1 according to the OECD guideline [19]. First, the standard soil (SS) contained sand and kaolin only (Sigma-Aldrich, St Louis, USA). This soil fulfilled composition and pH of an OECD artificial soil except for the *Sphagnum* peat portion, which was not included to avoid any sorption competition with CLD. The second soil (DARCO) was based on SS and supplemented with DARCO (Sigma-Aldrich, St Louis, USA) and the third one (ORBO) was based on SS and contained ORBO 32 (Sigma-Aldrich, St Louis, USA). In ORBO and DARCO soils, ACs were added to achieve 2% of the soil mass dry matter.

All three soils were spiked to attain 100 μg of CLD (Kepone, Supelco, Sigma Aldrich, Saint-Louis) per g of soil dry matter. CLD was spread over soil using an aqueous mixture of CLD (20:80; vol:vol; methanol:water). Then, all samples were thoroughly hand-mixed with a spatula during 10 minutes. Solvent traces were evaporated under an extractor hood overnight. Then, milliQ water was added to reach a 17.5% moisture content in each vial. Soil-samples were hand-mix thoroughly using a spatula during an additional 10-minute period. All the three soils were stored at 20°C in amber glass vials for a 3-week maturation period until the first day of exposure (day 1).

#### 2.3.3. Exposure of animals

All goat kids were exposed daily to one soil fraction according to their individual body weight. The day before exposure, the corresponding amount of soil (1 g of soil dry matter per 10 kg of body weight of kid) was incorporated in the centre of a moistened feed dough ball. The dough balls were fed individually to the kids daily after an overnight fasting period.

### 2.4. Sampling and analysis of biological samples

After 21 days of exposure, kids were anaesthetized by electronarcosis followed by immediate exsanguination. Perirenal adipose tissue was collected as the reference tissue usually collected in slaughterhouses to control CLD residues according to the French national surveillance plan [20]. The whole liver was also collected in view of the high concentrations of CLD in this tissue. Samples were stored at -20°C and freeze-dried.

The CLD Quantification was performed on both of these biological matrices using Liquid Chromatography-tandem Mass Spectrometry (LC-MS/MS) according to the method ANSES PBM Pest LSA-INS-0164 v5 [21], in the Departmental Analytical Laboratory of Morbihan (LDA 56, Saint-Ave, France). The limit of quantification (LOQ) was 2.0 μg CLD kg^−1^ in those matrices, and the limits of detection were less than 0.7 μg CLD per kg of the tested matrix.

The analytical method was initially developed by the French Agency for Food, Environmental and Occupational Health and Safety (ANSES, Maisons‐Alfort Laboratory for food safety) which is the national reference laboratory for this molecule in France. This methodology is the current regulatory analytical reference used in France [22] to control CLD in foodstuffs according to the maximum residue levels established in European regulation [23].

Prior to the extraction step, a ^13^C (^13^C_8_C_2_Cl_10_O, Azur-isotope, Marseille, France, 98% of purity) internal standard of CLD was added to subsamples. Briefly, biological matrices were extracted sequentially. (i) For adipose tissue, 0.5 g of sample were added to 3 ml of a mix of acetonitrile and dichloromethane 75:25 (v:v). After centrifugation (1 200 x *g*, 20 min at -20°C) the supernatant was extracted. This extraction was performed 2 times. Then, the solvent was evaporated at 40°C under a nitrogen flux until dry. A mix of 15ml of hexane/acetone 85:15 (v:v) was then added. (ii) For liver, a sample of 2 g was used. Ten mL of hexane:acetone 85:15 (v:v) was added to the sample, which was then grinded using Ultraturrax® (10 000 rpm, 1 min, S25N-10G). After mixing (Vortex^®^ apparatus) and centrifugation (750 x *g*, 3 min) the supernatant was collected.

An alkalinisation step followed by an acidification step were performed to obtain CLD hydrate and to reform CLD, as described elsewhere [24]. Analytical grade sodium hydroxide solution (addition of 5 mL of 0.5 M NaOH aqueous solution to supernatant). After a mixing procedure (Vortex^®^ apparatus) and centrifugation (750 x *g*, 3 min), the supernatant was collected. This step was repeated two times.

The resulting aqueous phase was washed with 5 mL hexane to eliminate fat. The supernatant was collected after centrifugation (750 x *g*, 3 min). CLD was reformed through acidification of the solution by means of sulfuric acid (5mL of 60% solution). A second extraction phase was carried out with hexane:acetone (5mL of 85:15 v/v), followed by mixing (Vortex^®^ apparatus) and centrifugation (750 x *g*, 3 min). This extraction step was repeated 3 times. The organic phase was then rinsed with 2mL of water, before being subsequently evaporated until dry and 1mL of methanol was then added.

Separation was achieved using a Phenomenex Aqua C18 column (150x2.0 mm 3 μm) and a precolumn Phenomenex Aqua C18 (4x3.0 mm). Two phases were used to perform the separation step: water with 0.1% formic acid (A) and methanol with 0.1% formic acid (B). Five μL of sample was injected per run and the flow was set to 200 μl/min. After separation, a rinse sequence using acetonitrile was performed. Quantitation by isotope-dilution was performed on API 5500. Parent ions (PI) and child ones (CI) were used to respectively quantify (507/427 Da; PI/CI) and qualify (509/429 Da; PI/CI) ^12^C CLD and quantify (517/436 Da; PI/CI) ^13^C CLD.

### 2.5. Data analyses

#### 2.5.1. Quality control of data acquisition and data set

LDA 56 works under the international standard ISO/IEC 17025:2005 (General requirements for the competence of testing and calibration laboratories). In addition, analyses were carried out in strict accordance with the COFRAC (the French Accreditation Committee) quality accreditation of the LDA 56 when applying the analytical methodology for animal foodstuff ANSES PEST LSA-INS-0164 [21]. This quality accreditation involves frequent interlaboratory tests. Uncertainty of this methodology was proven to be less than 20% for adipose tissue and fish [21].

Some values below LOQ were replaced by LOQ value (2.0 μg CLD kg^−1^ in tested matrices) in the data set.

#### 2.5.2. Tissues concentrations of CLD

In order to assess the impact of ACs on bioavailability of the contaminant an analysis of variances was performed. The experimental unit was the kid. Concentrations of CLD in adipose tissue and liver were compared between the three treatment groups (SS, ORBO, DARCO; n = 5 for each group) using the ANOVA procedure and the Tukey–Kramer post-hoc test of R version 3.2.3 (R Foundation for Statistical Computing, Vienna, Austria). Differences were considered significant at P<0.05.

#### 2.5.3. Relative bioavailability (RBA) factor calculation

Statistical analyses were carried out using R (version 3.2.3, R Foundation for Statistical Computing) on CLD concentration. Each kid was considered as an experimental unit. A 95% confidence interval of concentrations was calculated as described elsewhere [25].

The RBA was calculated by dividing “CLD-concentrations in one tissue (liver or adipose tissue) after one treatment (DARCO or ORBO)” by “CLD concentrations obtained in the same tissue after SS treatment (100% reference)”, adapted from a method previously described [16,17]. Linearity between the CLD-dose of exposure and CLD-concentrations in the organ was a prerequisite of this method [26]. This linearity was demonstrated previously for CLD in lambs [8]. Since lambs and goats have a similar physiology, the dose-response of CLD was not assessed in the present study.

The corresponding equation to calculate RBA of CLD after one AC-treatment in the liver is provided below:

$$RBA_{AC\;treatment;liver}=\frac{C{{}^{\circ}}_{AC\;treatment\;;\;liver}}{C{{}^{\circ}}_{SS\;treatment\;;\;iver}}$$

$$\begin{array}{l} RBA_{AC\;treatment;liver}:\;\mathrm{Relative}\;\mathrm{bioavailability}\;\mathrm{of}\;\mathrm{CLD}\;\mathrm{after}\;\mathrm{one}\;\mathrm{AC}-\mathrm{treatment}\;\mathrm{in}\;\mathrm{the}\;\mathrm{liver}\\ C{{}^{\circ}}_{AC\;treatment;liver}:\;\;\mathrm{CLD}\;\mathrm{concentration}\;\mathrm{in}\;\mathrm{liver}\;\mathrm{after}\;\mathrm{one}\;\mathrm{AC}\;\mathrm{treatment}\\ C{{}^{\circ}}_{SS\;treatment;liver\;}:\;\mathrm{CLD}\;\mathrm{concentration}\;\mathrm{in}\;\mathrm{liver}\;\mathrm{after}\;\mathrm{SS}\;\mathrm{treatment}: \end{array}$$

As each part of the division comprises uncertainties, a 95% confidence interval of the RBA factor was calculated via the propagation of uncertainty rule [27]. The corresponding equation is provided above:

$$\frac{\Delta_{RBA}}{RBA}=\frac{\Delta_{C{{}^{\circ}}_{AC\;treatment}}}{C{{}^{\circ}}_{AC\;treatment}}+\;\frac{\Delta_{C{{}^{\circ}}_{SS\;treatment}}}{C{{}^{\circ}}_{SS\;treatment}}$$

$$\begin{array}{l} \Delta_{RBA}:\;\;\mathrm{Relative}\;\mathrm{uncertainty}\;\mathrm{of}\;\mathrm{relative}\;\mathrm{bioavailability}\;\mathrm{after}\;\mathrm{one}\;\mathrm{AC}-\mathrm{treatment}\\ \;\mathrm{RBA}:\;\;\mathrm{Relative}\;\mathrm{bioavailability}\\ \Delta_{C{{}^{\circ}}_{AC\;treatment}}:\;\mathrm{Relative}\;\mathrm{uncertainty}\;\mathrm{of}\;\mathrm{concentration}\;\mathrm{found}\;\mathrm{in}\;\mathrm{one}\;\mathrm{tissue}\;\mathrm{after}\;\mathrm{one}\;\mathrm{AC}-\mathrm{treatment}\\ C{{}^{\circ}}_{AC\;treatment}:\;\mathrm{CLD}\;\mathrm{concentration}\;\mathrm{after}\;\mathrm{one}\;\mathrm{AC}\;\mathrm{treatment}\\ \Delta_{C{{}^{\circ}}_{SS\;treatment}}:\;\mathrm{Relative}\;\mathrm{uncertainty}\;\mathrm{of}\;\mathrm{CLD}\;\mathrm{concentration}\;\mathrm{found}\;\mathrm{in}\;\mathrm{one}\;\mathrm{tissue}\;\mathrm{after}\;\mathrm{SS}\;\mathrm{treatment}\\ C{{}^{\circ}}_{SS\;treatment}:\;\mathrm{CLD}\;\mathrm{concentration}\;\mathrm{found}\;\mathrm{in}\;\mathrm{one}\;\mathrm{tissue}\;\mathrm{after}\;\mathrm{SS}\;\mathrm{treatment} \end{array}$$

#### 2.5.4. Calculation of CLD retention by ACs during the digestive process

A factor of CLD retention by AC was determined as the ratio between concentrations obtained after SS treatment and concentrations obtained using ORBO and DARCO in the same biological matrix. In order to be conservative, this factor was minimized using the lowest and highest values of the 95^th^ confidence interval of concentrations of CLD. Details of the calculations are provided above for AC DARCO using concentrations of CLD in liver:

$$R_{DARCO;liver}=\frac{Min_{95}\;C{{}^{\circ}}_{liver\;}DARCO}{Max_{95}C{{}^{\circ}}_{liver\;}SS}$$

$$\begin{array}{l} \;R_{DARCO;liver\;}:\;\;\mathrm{factor}\;\mathrm{of}\;\mathrm{CLD}\;\mathrm{retention}\;\mathrm{by}\;\mathrm{AC}\;\mathrm{DARCO}\;\mathrm{using}\;\mathrm{concentrations}\;\mathrm{of}\;\mathrm{CLD}\;\mathrm{in}\;\mathrm{liver}\\ Min_{95}\;C{{}^{\circ}}_{liver\;}DARCO:\;\mathrm{lowest}\;\mathrm{value}\;\mathrm{of}\;95\mathrm{th}\;\mathrm{confidence}\;\mathrm{interval}\;\mathrm{of}\;\mathrm{CLD}\;\mathrm{concentration}\;\mathrm{in}\;\mathrm{liver}\;\left(\mathrm{statistical}\;\mathrm{model}\;\mathrm{explained}\;\mathrm{in}\;2.5.2\right)\;\mathrm{for}\;\mathrm{SS}\;\mathrm{with}\;\mathrm{DARCO}\;\mathrm{treatment}\;\;\;Max_{95}\;C{{}^{\circ}}_{liver\;}SS:\;\mathrm{highest}\;\mathrm{value}\;\mathrm{of}\;95\mathrm{th}\;\mathrm{confidence}\;\mathrm{interval}\;\mathrm{of}\;\mathrm{CLD}\;\mathrm{concentration}\;\mathrm{in}\;\mathrm{liver}\;\left(\mathrm{statistical}\;\mathrm{model}\;\mathrm{explained}\;in\;2.5.2\right)\;for\;SS\;treatment \end{array}$$

## 3. Results & discussion

Daily ingestion of feed was unaffected by treatment during the exposure period (16 ± 3 g.kg^-1^.day^-1^, P>0.10). Moreover, no significant effect on daily weight gain (160 ± 50 g.day^-1^, mean ± SD, P>0.10) could be discerned at the end of the exposure period.

### 3.1. Concentrations of CLD in biological matrices

**Fig 2:**
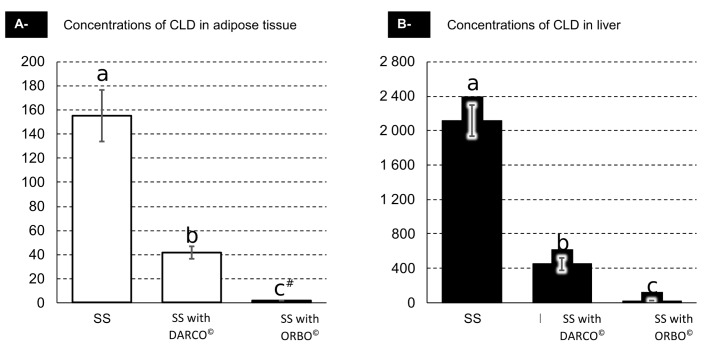
Concentrations of CLD in biological matrices (ng of CLD per g of DM). Concentrations of CLD are expressed in ng.g^-1^ of DM. Values correspond to the mean ± SD. Mean values with different superscript letters (a, b, c) were statistically different (P<0.05). Statistical analysis was performed using the two-way ANOVA procedure of R software and a Tukey post-hoc test. Two effects were used in the model: Organ and Treatment. Both effects were significant (p<0.0001) and RMSE = 12.84. (n = 5). RMSE: Root means square error. #: values are below limit of quantification (LOQ).

Concentrations of CLD in biological matrices showed important differences between the treatment groups (*cf* Fig 2). As expected, the highest CLD concentrations were obtained in the SS group (without AC) for both matrices: 155 ± 22 ng/g of DM (adipose tissue; mean ± SD) and 2110 ± 180 ng/g of DM (liver; mean ± SD). Intermediate ones were obtained in DARCO treated animals: 41.3 ± 5.2 ng/g of DM (adipose tissue; mean ± SD); 449 ± 71 ng/g of DM (liver; mean ± SD), and the lowest CLD concentrations in ORBO treated animals with non-quantifiable levels for adipose tissue (<2 ng/g DM) and 24.0 ± 2.4 ng/g DM (liver; mean ± SD).

The ANOVA analyses revealed a major treatment effect (*cf* Fig 2) for both biological matrices (p<0.0001 for both tissues). Further investigation using a post-hoc Tukey test demonstrated that all treatment groups were distinct from each other (p<0.01).

Within each treatment group, CLD concentrations in liver were > 10-fold higher than those of adipose tissue. These differences between both organs are consistent with previous observations in rodent [28], lambs [8], pigs [9] and humans [29]. This particular affinity of CLD to liver could be explained by non-clearly identified proteins which readily bound CLD [30].

### 3.2. Effects of activated carbon on the CLD RBA

The RBA factors were calculated in order to estimate the CLD bioavailable fraction after the following treatments: ORBO or DARCO. Compared to SS, the DARCO treatment showed RBAs of 26.6% and 21.2% (respectively from adipose tissue and liver) and the ORBO treatment revealed RBAs of 1.3% (maximum value from adipose tissue) and 1.1% (liver). Similar RBA factors were obtained from adipose tissue and liver with the exception of ORBO for adipose tissue, where just a maximum value could be assessed (as all concentrations were below LOQ). Thus, SS with DARCO.

Statistical analyses were performed to obtain 95% confidence interval as presented in Table 2. To assess the efficiency of sequestration by ACs during the digestive processes, a factor of sequestration was calculated. DARCO exhibited a reduction of 53.4% (adipose tissue) and 67.2% (liver tissue). ORBO displayed a greater reduction of 98.2% (adipose tissue; base on LOQ) and 98.4% (liver) (Table 3).

**Table 2:** Relative bioavailability factors of CLD in biological matrices (% and 95% confidence interval).

	Adipose Tissue	Liver
SS with DARCO	26,6% [12.0% -41.2%]	21.2 [11.5%–30.9%]
SS with ORBO	1,3% [NA]	1.1% [0.7%–1.5%]

Values in brackets indicates 95% confidence interval (2,7xSE). SE were calculated via propagation of errors formula. (n = 5)

**Table 3:** Reduction factor (%, based on 95th confidence interval).

	Adipose Tissue	Liver
SS with DARCO	53.4%	67.2%
SS with ORBO	98.2%	98.4%

Reduction factor are calculated as described in material and method section.

In the French West Indies contaminated areas, CLD concentrations in soil vary between 0.011–52.1 mg.kg^−1^ of dry soil, as reported by Le Villain et *al* (2012) using the database of the Crop Protection Service (French Department of Agriculture) [31]. Mean and median values of CLD concentrations are respectively 2.1 and 1.1 mg.kg^-1^ [31]. Thus, the level of CLD in soil (50 μg.g^-1^) used in this study was similar to the maximal value within this database (52.1 mg.kg^-1^). Thus, the saturation of AC medium by CLD should not be a limiting process if in field conditions.

The surface properties and textural characteristics of ACs drive the extent of adsorption of CLD [15,32]. In particular, the surface specific activity (SSA) is believed to be positively correlated to its binding potentiality [33]. ACs used in the present study are microporous media, even if ORBO presents a greater part of microporosity (80%) than DARCO (56%). The differences of the precursor materials of those ACs (coconut shell and lignite for ORBO and DARCO ACs, respectively) lead to different structures of porosity. Upon supplementation, this nanoporous material binds tightly halogenated compounds present in soil [33–35]. In addition, this structure could explain the limitation of the CLD desorption in the digestive chyme [36]. This result is in line with previous observations on polychlorinated biphenyls [16,36]. This process referred to as “physical trapping” of organic pollutants is a very limiting step towards uptake of CLD by animal [36]. The effectiveness of this trapping appears greater for ORBO than DARCO as shown by the RBA factors. This difference can be explained by the higher SSA displayed by ORBO (1126 versus 794 m^2^ g^-1^) leading to an increase of its CLD-binding capacity. Such a high level of microporosity could explain its capacity to physically trap CLD.

## 4. Conclusion

Both ACs efficiently retained CLD during the digestive processes of goat kids. When added to artificial soil, these ACs allowed the significant limitation of the bioavailable fraction, ranging from 64% to 98% for DARCO and ORBO respectively. These differences were linked to the specific SSA of the two ACs. This study performed on artificial soils is a very promising step before investigating the effects of ACs in on-field contaminated soils. Further investigations will be developed in order to assess the sequestration-efficiency of different levels of ACs or CLD- historically contaminated soils in the French West Indies.

## Supporting information

S1 Table Concentrations of CLD in biological matrices in each individual (ng of CLD per g of DM). Concentrations of CLD are expressed in ng.g^-1^ of DM.
#: values are below limit of quantification (LOQ).
(DOCX)

## References

[1] Le Déaut J-Y, Procaccia C. Impacts de l’utilisation de la chlordécone et des pesticides aux Antilles: bilan et perspectives d’évolution [Internet]. Sénat; 2009 pp. 1–223. Available: internal-pdf://2212 Le Deaul 2009-2841645057/2212 Le Deaul 2009.pdf

[2] Cabidoche YM, Achard R, Cattan P, Clermont-Dauphin C, Massat F, Sansoulet J. Long-term pollution by chlordecone of tropical volcanic soils in the French West Indies: A simple leaching model accounts for current residue. Environ Pollut. 2009;157: 1697–1705. doi: 10.1016/j.envpol.2008.12.015 19167793 10.1016/j.envpol.2008.12.015

[3] Faroon O, Kueberuwa S. Toxicological Profile for Mirex and Chlordecone. ATSDR Agency Toxic Subst Dis Regist. 1995; 1–333. 37075159

[4] Coat S, Monti D, Legendre P, Bouchon C, Massat F, Lepoint G. Organochlorine pollution in tropical rivers (Guadeloupe): Role of ecological factors in food web bioaccumulation. Environ Pollut. 2011;159: 1692–1701. doi: 10.1016/j.envpol.2011.02.036 21440344 10.1016/j.envpol.2011.02.036

[5] Dromard CR, Bodiguel X, Lemoine S, Bouchon-Navaro Y, Reynal L, Thouard E, et al Assessment of the contamination of marine fauna by chlordecone in Guadeloupe and Martinique (Lesser Antilles). Environ Sci Pollut Res. 2016;23: 73–80. doi: 10.1007/s11356-015-4732-z 25994274 10.1007/s11356-015-4732-z

[6] Jondreville C, Fournier A, Mahieu M, Feidt C, Archimède H, Rychen G. Kinetic study of chlordecone orally given to laying hens (Gallus domesticus). Chemosphere. 2014;114: 275–281. doi: 10.1016/j.chemosphere.2014.05.008 25113213 10.1016/j.chemosphere.2014.05.008

[7] Jondreville C, Lavigne A, Jurjanz S, Dalibard C, Liabeuf J-M, Clostre F, et al Contamination of free-range ducks by chlordecone in Martinique (French West Indies): A field study. Sci Total Environ. 2014;493: 336–341. doi: 10.1016/j.scitotenv.2014.05.083 24951891 10.1016/j.scitotenv.2014.05.083

[8] Jurjanz S, Jondreville C, Mahieu M, Fournier A, Archimède H, Rychen G, et al Relative bioavailability of soil-bound chlordecone in growing lambs. Environ Geochem Health. 2014;36: 911–917. doi: 10.1007/s10653-014-9608-5 24729076 10.1007/s10653-014-9608-5

[9] Bouveret C, Rychen G, Lerch S, Jondreville C, Feidt C. Relative bioavailability of tropical volcanic soil-bound Chlordecone in piglets. J Agric Food Chem. 2013;61: 9269–9274. doi: 10.1021/jf400697r 23992462 10.1021/jf400697r

[10] Jondreville C, Bouveret C, Lesueur-Jannoyer M, Rychen G, Feidt C. Relative bioavailability of tropical volcanic soil-bound chlordecone in laying hens (Gallus domesticus). Environ Sci Pollut Res. 2013;20: 292–299. doi: 10.1007/s11356-012-1010-1 22684877 10.1007/s11356-012-1010-1

[11] Choi Y, Cho Y-M, Luthy RG. In Situ Sequestration of Hydrophobic Organic Contaminants in Sediments under Stagnant Contact with Activated Carbon. 1. Column Studies. Environ Sci Technol. 2014;48: 1835–1842. doi: 10.1021/es403335g 24083415 10.1021/es403335g

[12] Hilber I, Bucheli TD. Activated Carbon Amendment to Remediate Contaminated Sediments and Soils: A Review. Glob Nest J. 2010;12: 305–317.

[13] Jakob L, Hartnik T, Henriksen T, Elmquist M, Brändli RC, Hale SE, et al PAH-sequestration capacity of granular and powder activated carbon amendments in soil, and their effects on earthworms and plants. Chemosphere. 2012;88: 699–705. doi: 10.1016/j.chemosphere.2012.03.080 22546631 10.1016/j.chemosphere.2012.03.080

[14] Perelo LW. Review: In situ and bioremediation of organic pollutants in aquatic sediments. J Hazard Mater. 2010;177: 81–89. doi: 10.1016/j.jhazmat.2009.12.090 20138425 10.1016/j.jhazmat.2009.12.090

[15] Gaspard S, Durimel A, Passé-Coutrin T, Jeanne-Rose V. Activated carbons from *plantae* and marine biomass for water treatment In: Gaspard S, Ncibi C, editors. Biomass for sustainable applications-Energy production and storage and pollution remediation. Royal society of chemistry *in press*, United Kingdom; 2014.

[16] Delannoy M, Rychen G, Fournier A, Jondreville C, Feidt C. Effects of condensed organic matter on PCBs bioavailability in juvenile swine, an animal model for young children. Chemosphere. 2014;104: 105–112. doi: 10.1016/j.chemosphere.2013.10.072 24289980 10.1016/j.chemosphere.2013.10.072

[17] Wittsiepe J, Erlenkämper B, Welge P, Hack A, Wilhelm M. Bioavailability of PCDD/F from contaminated soil in young Goettingen minipigs. Chemosphere. 2007;67: S355–S364. doi: 10.1016/j.chemosphere.2006.05.129 17223170 10.1016/j.chemosphere.2006.05.129

[18] Brunauer S, Emmett PH, Teller E. Adsorption of gases in multimolecular layers. J Am Chem Soc. 1938;60: 309–319.

[19] OECD. Test n° 207 earthworm acute toxicity test. Section 4: effects on biotic systems. Paris: OECD Publishing; 1984. Available: http://www.oecd.org/dataoecd/54/61/38972970.pdf

[20] French Ministry of Agriculture. DGAL/SDPAL/2016-377 [Internet]. Paris: French Ministry of Agriculture; 2016 Feb. Report No.: N° NOR AGRG1507402N. Available: https://info.agriculture.gouv.fr/gedei/site/bo-agri/instruction-2015-266

[21] ANSES. Méthode de dosage des résidus de chlordécone dans les denrées d’origine animale. Maisons-Alfort, France: ANSES; 2015 Sep. Report No.: ANSES PBM Pest LSA-INS-0164-Version 05.

[22] French Ministry of Agriculture. DGAL/SDPAL/2015-266 [Internet]. Paris: French Ministry of Agriculture; 2015 Mar. Report No.: N° NOR AGRG1507402N. Available: https://info.agriculture.gouv.fr/gedei/site/bo-agri/instruction-2015-266

[23] Commission of the European Communities. Commission Regulation (EC) No 839/2008 of 31 July 2008 amending Regulation (EC) No 396/2005 of the European Parliament and of the Council as regards Annexes II, III and IV on maximum residue levels of pesticides in or on certain products [Internet]. Regulation (EC), 839/2008 Jul 31, 2008 pp. 82–89. Available: http://data.europa.eu/eli/reg/2008/839/oj

[24] Bordet F, Thieffinne A, Mallet J, Heraud F, Blateau A, Inthavong D. In-house validation for analytical methods and quality control for risk evaluation of chlordecone in food. Int J Environ Anal Chem Intern J Env Anal Chem. 2007;87: 985–998.

[25] Shafer DS, Zhang Z. Introductory statistics [Internet]. Saylor.org Academy; 2013. Available: https://www.saylor.org/site/textbooks/Introductory%20Statistics.pdf

[26] Littell RC, Henry PR, Lewis AJ, Ammerman CB. Estimation of relative bioavailability of nutrients using SAS procedures. J Anim Sci. 1997;75: 2672–2683. 9331869 10.2527/1997.75102672x

[27] Ku HH. Notes on the use of propagation of error formulas. J Res Natl Bur Stand. 1966;70 Available: http://nvlpubs.nist.gov/nistpubs/jres/70C/jresv70Cn4p263_A1b.pdf

[28] Egle JL Jr, Fernandez SB, Guzelian PS, Borzelleca JF. Distribution and excretion of chlordecone (kepone) in the rat. Drug Metab Dispos. 1978;6: 91–95. 74312

[29] Cohn WJ, Boylan JJ, Blanke RV, Fariss MW, Howell JR, Guzelian PS. Treatment of chlordecone (Kepone) toxicity with cholestyramine. Results of a controlled clinical trial. N Engl J Med. 1978;298: 243–248. doi: 10.1056/NEJM197802022980504 74014 10.1056/NEJM197802022980504

[30] Soine PJ, Blanke RV, Guzelian PS, Schwartz CC. Preferential binding of chlordecone to the protein and high density lipoprotein fractions of plasma from humans and other species. J Toxicol Environ Health. 1982;9: 107–118. doi: 10.1080/15287398209530146 6174734 10.1080/15287398209530146

[31] Levillain J, Cattan P, Colin F, Voltz M, Cabidoche Y-M. Analysis of environmental and farming factors of soil contamination by a persistent organic pollutant, chlordecone, in a banana production area of French West Indies. Agric Ecosyst Environ. 2012;159: 123–132. doi: 10.1016/j.agee.2012.07.005

[32] Durimel A, Altenor S, Miranda-Quintana R, Couespel Du Mesnil P, Jauregui-Haza U, Gadiou R, et al pH dependence of chlordecone adsorption on activated carbons and role of adsorbent physico-chemical properties. Chem Eng J. 2013;229: 239–249. doi: 10.1016/j.cej.2013.03.036

[33] Ahmad M, Rajapaksha AU, Lim JE, Zhang M, Bolan N, Mohan D, et al Biochar as a sorbent for contaminant management in soil and water: A review. Chemosphere. 2014;99: 19–33. doi: 10.1016/j.chemosphere.2013.10.071 24289982 10.1016/j.chemosphere.2013.10.071

[34] Beesley L, Moreno-Jiménez E, Gomez-Eyles JL, Harris E, Robinson B, Sizmur T. A review of biochars’ potential role in the remediation, revegetation and restoration of contaminated soils. Environ Pollut. 2011;159: 3269–3282. doi: 10.1016/j.envpol.2011.07.023 21855187 10.1016/j.envpol.2011.07.023

[35] Yavari S, Malakahmad A, Sapari NB. Biochar efficiency in pesticides sorption as a function of production variables—a review. Environ Sci Pollut Res. 2015;22: 13824–13841. doi: 10.1007/s11356-015-5114-2 26250816 10.1007/s11356-015-5114-2

[36] Delannoy M, Schwarz J, Fournier A, Rychen G, Feidt C. Effects of Standard Humic Materials on Relative Bioavailability of NDL-PCBs in Juvenile Swine. PLoS ONE. 2014;9: e115759 doi: 10.1371/journal.pone.0115759 25549096 10.1371/journal.pone.0115759 PMC4280112

