# Supplementary material for: Activated carbon, a useful medium to bind chlordecone in soil and limit its transfer to growing goat kids
Source: PLoS One. 2017 Jul 19;12(7):e0179548. doi: 10.1371/journal.pone.0179548 (PMC5516976; doi:10.1371/journal.pone.0179548)
Supplement: S1 Table — Concentrations of CLD are expressed in ng.g-1 of DM. #: values are below limit of quantification (LOQ). (DOCX) [file pone.0179548.s001.docx]

***Activated carbon, a useful medium to bind chlordecone in soil and limit its transfer to growing goat kids***

**Sarah Yehya^1,2^, Matthieu Delannoy^1^, Agnès Fournier^1^, Moomen Baroudi^2^, Guido Rychen^1^, Cyril** **Feidt^1^.**

1 : Université de Lorraine, INRA USC 340, UR AFPA, 2 avenue de la Forêt de Haye TSA 40602, 54 518 Vandœuvre-lès-Nancy, France

2 : Lebanese University – Faculty of Public Health-Section III, L.S.E.E., Tripoli, Lebanon

*Corresponding author: [matthieu.delannoy@univ-lorraine.fr](mailto:matthieu.delannoy@univ-lorraine.fr) (MD)

| Kid goats individual | Exposure medium | CLD concentrations in adipose tissue (ng.g^-1^ adipose tissue) | CLD concentration in liver (ng.g^-1^ in liver) |
| --- | --- | --- | --- |
| 192 | SS with DARCO^©^ | 47,9 | 329 |
| 194 | SS with DARCO^©^ | 44,5 | 472 |
| 197 | SS with DARCO^©^ | 36 | 485 |
| 200 | SS with DARCO^©^ | 42 | 508 |
| 195 | SS with DARCO^©^ | 36,3 | 449 |
| 189 | SS with ORBO ^©^ | <2^#^ | 20 |
| 198 | SS with ORBO ^©^ | <2^#^ | 24,7 |
| 188 | SS with ORBO ^©^ | <2^#^ | 23,9 |
| 196 | SS with ORBO ^©^ | <2^#^ | 25,3 |
| 185 | SS with ORBO ^©^ | <2^#^ | 26,1 |
| 193 | Standard soil | 146,9 | 1970 |
| 199 | Standard soil | 140,4 | 2130 |
| 186 | Standard soil | 193,2 | 2400 |
| 187 | Standard soil | 143,7 | 1950 |
| 190 | Standard soil | 152,7 | 2120 |

**Table S1. Concentrations of CLD in biological matrices in each individual (ng of CLD per g of DM)**

Concentrations of CLD are expressed in ng.g^-1^ of DM.

#: values are below limit of quantification (LOQ).
